# Exploring the therapeutic role of early heparin administration in ARDS management: a MIMIC-IV database analysis

**DOI:** 10.1186/s40560-024-00723-5

**Published:** 2024-02-26

**Authors:** Ling-Xi Xiao, De Liang Zhu, Juan Chen, Jing Lv, Mei-Jun Liu, Xue Dai, Dao-Xin Wang, Wang Deng

**Affiliations:** 1 Department of Pulmonary and Critical Care Medicine, Second Affiliated Hospital of Chongqing Medical University, 76 Linjiang Road, Yuzhong District, Chongqing, 400010 China; 2 Department of Gastrointestinal Surgery, China-Japan Union Hospital of Jilin University, No. 126, Xiantai Street, Changchun, 130033 China

**Keywords:** Acute respiratory distress syndrome, Heparin, Propensity score, Intensive care units, Mortality, MIMIC-IV database

## Abstract

**Background:**

Acute respiratory distress syndrome (ARDS) is a severe respiratory condition characterized by a high mortality rate, the management of which relies on supportive care and a profound understanding of its pathophysiology. Heparin, with its anticoagulant and potential anti-inflammatory properties, offers a new therapeutic opportunity for the treatment of ARDS.

**Methods:**

In this retrospective cohort study, we examined the MIMIC-IV database for ARDS patients who received prophylactic heparin within the first 72 h of ICU admission. Employing propensity score matching and inverse probability weighting (IPW) analysis, we evaluated the impact of early heparin use on patient outcomes, focusing on mortality rates.

**Results:**

Patients who received prophylactic heparin had a significantly lower in-hospital mortality rate compared to those who did not (13.55% vs 17.93%, HR = 0.71, 95% CI: 0.54–0.93, *P* = 0.012). This result remained significant after propensity score matching (12.75% vs 17.93%, HR = 0.65, 95% CI 0.47–0.90, *P* = 0.010). Analysis using five different statistical models indicated that early use of heparin significantly reduced the in-hospital mortality rate, with HR = 0.669 (95% CI 0.487–0.919, *P* = 0.013) in the doubly robust model without balanced covariates; HR = 0.705 (95% CI 0.515–0.965, *P* = 0.029) with all covariates considered; HR = 0.660 (95% CI 0.491–0.888, *P* = 0.006) in the propensity score (IPW) model; HR = 0.650 (95% CI 0.470–0.900, *P* = 0.010) in the propensity score matching model; and HR = 0.706 (95% CI 0.536–0.930, *P* = 0.013) in the multivariate Cox regression model. Secondary outcomes indicated that heparin use was also associated with reduced mortality rates at 60 days, and 90 days.

**Conclusion:**

This research highlights that early prophylactic administration of heparin may substantially lower mortality in ARDS patients. These findings underscore the potential of heparin as a key component in the management of ARDS, offering a new perspective and novel strategies for clinical treatment.

**Supplementary Information:**

The online version contains supplementary material available at 10.1186/s40560-024-00723-5.

## Background

Acute respiratory distress syndrome (ARDS) is characterized by acute respiratory failure caused by extensive pulmonary inflammation and edema, often culminating in high fatality rates [1]. Its etiology is multifaceted, encompassing both infectious and non-infectious factors. As a life-threatening condition, the mortality rate of ARDS varies depending on the initial severity, ranging approximately from 35 to 45% [2]. Current treatment strategies for ARDS primarily rely on supportive care, with no effective targeted pharmaceutical therapies available. In the pathophysiological progression of ARDS, the disruption of coagulation and inflammatory responses plays a pivotal role. Endothelial cells shift from an antithrombotic and anti-inflammatory phenotype to an activated state that promotes thrombosis and inflammation, a transition crucial to the disease's progression [3]. Specifically, an increase in procoagulant activity within the pulmonary alveolar membranes, coupled with a decrease in fibrinolytic activity, leads to the accumulation of fibrin, changes that may support gas exchange and tissue repair but could also induce further pulmonary dysfunction and fibrosis [4].

Amidst this backdrop, heparin has garnered considerable attention for its multifaceted biological effects. As an anticoagulant, heparin limits the deposition of fibrin within the alveoli, alleviating the progression of pulmonary injury [5]. Moreover, heparin possesses anti-inflammatory properties, playing a key role in mitigating ARDS's inflammatory response by inhibiting chemotactic factors and cytokines, impeding the migration of leukocytes, and preventing the activation of the complement system [6]. Additionally, heparin can bind to specific bacteria and viruses, limiting the spread of infections [7], and modulating inflammatory pathways, reducing the risk of bronchospasm [8, 9], the formation of microvascular thrombosis, and endothelial damage [10, 11]. Heparin also facilitates the breakdown of DNA/histone complexes and neutrophil extracellular traps (NETs), reducing the viscosity and elasticity of airway secretions and neutralizing the effects of cytotoxic proteins such as histones [12, 13], thereby offering new strategic directions for the treatment of ARDS.

Based on this understanding, multicenter, double-blind trials such as the CHARLI study have suggested that nebulized heparin may limit lung injury by inhibiting pulmonary fibrin deposition and potentially enhance the recovery process of ARDS patients [14, 15]. Animal studies also support this notion, where subcutaneous injection of heparin significantly attenuated pulmonary injury and inflammation in ARDS model mice, thereby improving survival rates [16].

This study systematically evaluates the effects of early heparin use in ARDS patients within the MIMIC-IV v2 database to explore its potential impact on reducing mortality and shortening hospital stays. Our hypothesis posits that early heparin use, particularly in patients clinically diagnosed with ALI/ARDS, can reduce mortality by modulating coagulation and inflammatory responses. This research may offer a new perspective on the management of ARDS and novel strategies for clinical treatment.

## Methods and materials

### Data source and study population

This retrospective cohort study was conducted through an analysis of patient data extracted from the MIMIC-IV v2 database [17]. The MIMIC-IV database, a public resource that Beth Israel Deaconess Medical Center and the Massachusetts Institute of Technology jointly maintain, encompasses de-identified medical records of over 70,000 patients who received critical care at BIDMC between 2008 and 2019. This dataset provides comprehensive clinical information on patients, including laboratory test results, therapeutic interventions, medication usage, diagnostic codes, physiological parameters, and other medical interventions, offering a rich repository for patient-centric research.

### Inclusion and exclusion criteria

This study encompassed all patients who were definitively diagnosed with acute respiratory distress syndrome (ARDS) based on the Berlin criteria, aged 18 years or older, and had an intensive care unit (ICU) stay exceeding 48 h. Eligibility for inclusion in the analysis mandated the initiation of prophylactic heparin therapy within 72 h of ICU admission. In our study design, we excluded patients administered alternative anticoagulants, including rivaroxaban, warfarin, enoxaparin, argatroban, and fondaparinux. Additionally, cases utilizing heparin for purposes other than prophylaxis, such as for parenteral nutrition or anticoagulation in cardiac pacemaker therapy, were also excluded. Furthermore, patients receiving therapeutic doses of heparin were omitted from this study. The Berlin definition is as follows [18]: (1) acute onset of respiratory symptoms; (2) bilateral opacities on chest imaging; (3) partial pressure of arterial oxygen (PaO_2_) to fraction of inspired oxygen (FiO_2_) ratio < 300 mmHg with a minimum positive end-expiratory pressure (PEEP) of ≥ 5 cmH_2_O; and (4) absence of heart failure.

### Data collection and processing

Data collected included: (1) demographic characteristics such as gender, age, and ethnicity; (2) vital indicators, such as heart rate, body temperature, mean arterial pressure, and oxygen saturation (Spo2), during the first 24 h following ICU admission; (3) scoring systems evaluated in the first 24 h of ICU stay, including SOFA score, SAPS II score, and OASIS score; (4) comorbidities diagnosed via ICD-9 or ICD-10 codes, such as malignancy, diabetes, sepsis, chronic obstructive pulmonary disease (COPD), hypertension, acute pancreatitis, and acute renal failure; (5) therapeutic interventions such as the use of vasopressors, mechanical ventilation, renal replacement therapy (RRT), and continuous renal replacement therapy (CRRT). Prophylactic use of heparin was recorded in the medical orders, administered subcutaneously within 72 h of ICU admission, at a dose of 5000 units per administration. SQL queries and Navicat Premium software were used to extract data from the MIMIC-IV database. SPSS v28.0, Stata v17, R v4.3.1 and Python 3.12 were used for analysis.

### Outcome measures

The primary outcome measure in this study was the in-hospital mortality rate of patients. Secondary outcomes encompassed mortality rates at various intervals, specifically at 7 days, 14 days, 28 days, 60 days, and 90 days. Additionally, the length of stay in the Intensive Care Unit (ICU) and the overall duration of hospitalization were also evaluated as secondary outcomes.

### Statistical methods

Employing the Shapiro–Wilk test to examine whether continuous variables conform to a normal distribution. Depending on the distribution's normality, baseline characteristics were shown as means ± standard deviation or as the median and interquartile range (IQR). Counts and percentages were used to express categorical variables. Baseline characteristics between groups were compared using t-tests, Chi-square tests, Mann–Whitney *U* tests.

Propensity scores (PS) were calculated using Gradient Boosting Models (GBM) to estimate the probability of receiving early heparin treatment. Propensity score matching (PSM) was then performed to create matched cohorts for comparison based on baseline characteristics, with standardized mean differences (SMDs) used to assess match quality, where SMD > 0.1 indicated potential imbalance [19]. After matching, the balance was assessed through *P*-values, with *P* < 0.05 indicating a significant imbalance.

This study employed a multivariable Cox regression model to estimate risk factors associated with patient mortality. Specifically, we conducted a detailed assessment of the relationship between the dosage of prophylactic heparin used within the first 72 h and mortality rates. Additionally, to more accurately analyze the association between heparin use and patient outcomes, this research also utilized propensity scores (PS) derived from Gradient Boosting Machines (GBM). These propensity scores were then applied in dual robust analysis and Inverse Probability Weighting (IPW) methods to evaluate the effects of heparin usage [20].

To evaluate the potential impact of unmeasured confounding factors, an *E*-value analysis will be conducted. This analysis aims to estimate the magnitude of confounding effect that would be required to nullify the observed association between heparin use and mortality rates [21, 22].

Subgroup analysis was performed based on age, oxygenation index, respiratory rate, blood pressure, heart rate, and scoring systems. In subgroup analysis, multivariable Cox regression models were used to adjust for all the aforementioned variables, and the potential interaction between heparin use and variables specific to subgroups was evaluated.

The objective of this study was to thoroughly investigate the potential impact of different routes of administration on patient outcomes. To this end, a multivariable Cox regression model was utilized to analyze the effect of heparin administration via different routes and treatment dosages on the in-hospital mortality rate of patients.

## Results

### Patient characteristics

**Fig. 1:**
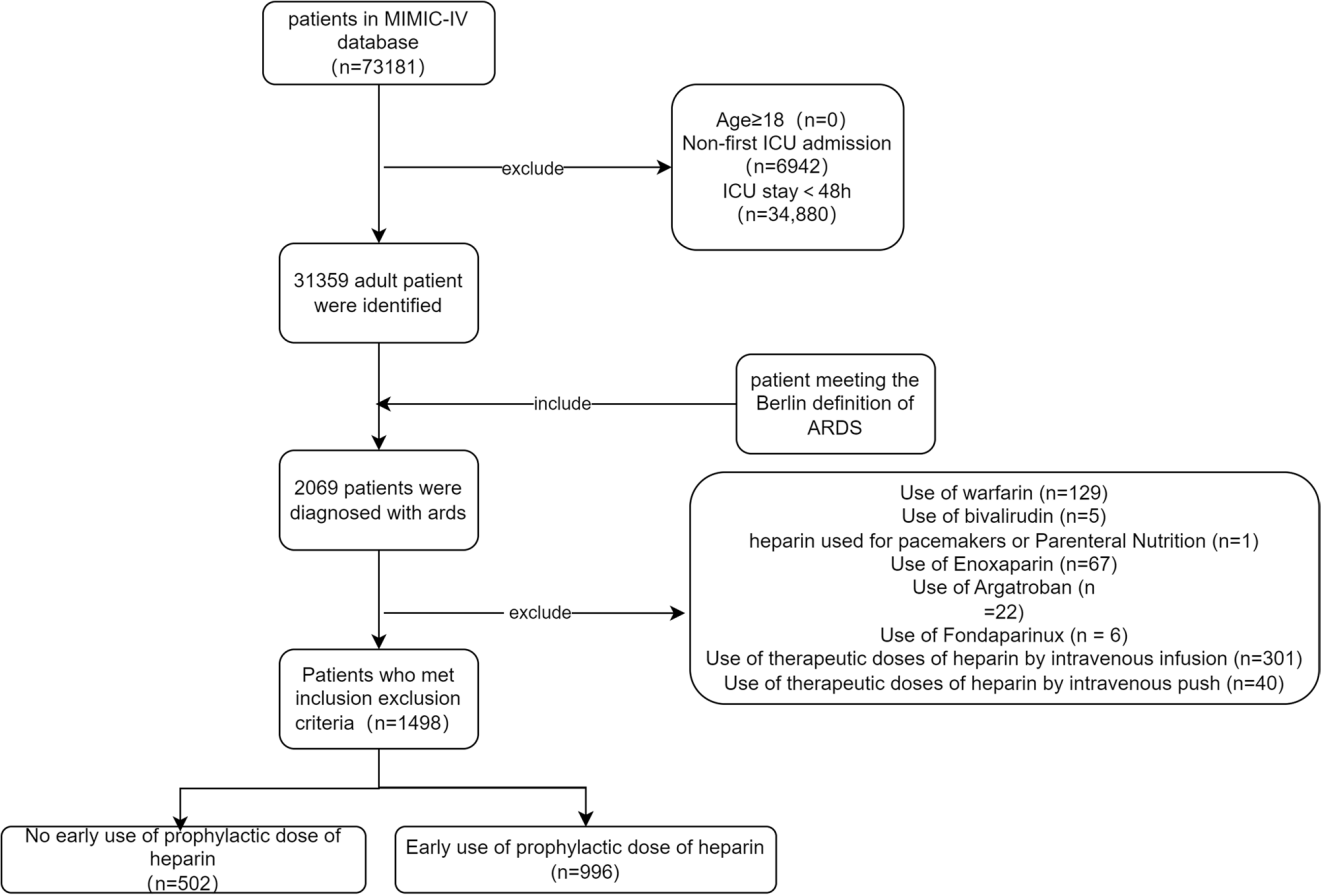
Enrollment flowchart for the selection of ARDS patient cohorts

**Fig. 2:**
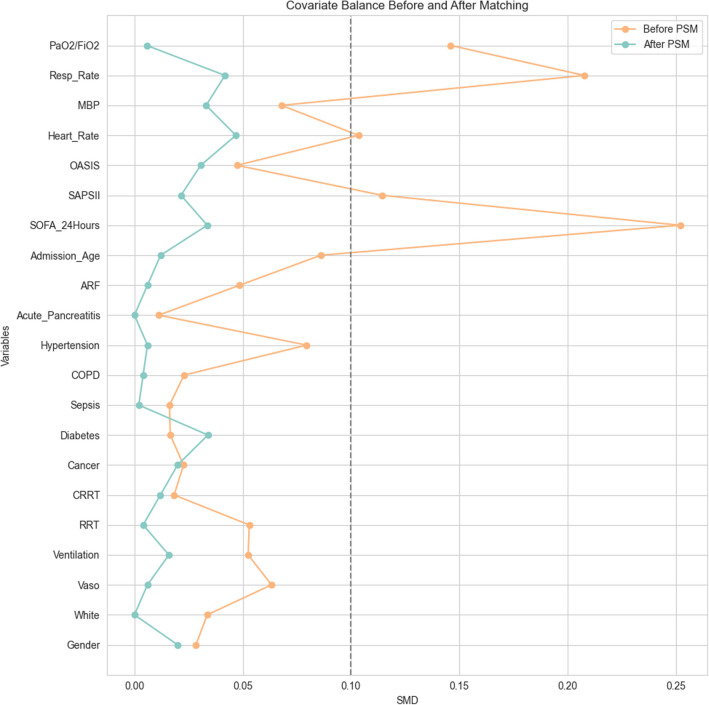
Pre- and post-propensity score matching the difference of baseline characteristics between the two groups

Following an analysis of 73,181 patients in the MIMIC-IV database, 1498 patients met the inclusion and exclusion criteria and were included in the study. The selection process, depicted in Fig. 1, divided the patients into two groups: 502 non-early heparin users and 996 early heparin users, to assess the impact of early heparin use on clinical outcomes. Baseline characteristic analysis revealed significant differences between the groups in various clinical parameters including age distribution, prevalence of hypertension, incidence of Acute Renal Failure (ARF), SOFA and SAPS II scores, heart rate, blood pressure, respiratory rate, use of vasopressors, and the application of renal replacement therapy (RRT). After Propensity Score Matching (PSM), the two groups achieved a good balance in these baseline features, with all Standardized Mean Differences (SMDs) being < 0.1, as shown in Table 1. This balance post-PSM is further illustrated in Fig. 2.

**Table 1:** Baseline characteristics of ARDS patient cohorts with absolute standardized mean differences pre- and post-matching

Characteristic	Before PSM					After PSM				
	All patients (*n* = 1498)	Non-heparin (*n* = 502)	Early heparin users (*n* = 996)	*P* value	SMD	All patients (*n* = 1004)	Non-heparin (*n* = 502)	Early heparin users (*n* = 502)	*P* value	SMD
Admission_age	65.0 [55.0, 77.0]	66.0 [56.0, 77.0]	65.0 [54.0, 77.0]	0.13	0.0861	66.0 [56.0, 77.0]	66.0 [56.0, 77.0]	65.0 [55.0, 78.0]	0.756	0.0121
Gender (female)	640 (42.72%)	205 (40.84%)	435 (43.67%)	0.321	0.0284	420 (41.83%)	205 (40.84%)	215 (42.83%)	0.565	0.0199
White	1002 (66.89%)	347 (69.12%)	655 (65.76%)	0.213	0.0336	694 (69.12%)	347 (69.12%)	347 (69.12%)	1	0
Cancer	234 (15.62%)	86 (17.13%)	148 (14.86%)	0.286	0.0227	182 (18.13%)	86 (17.13%)	96 (19.12%)	0.461	0.0199
Diabetes	464 (30.97%)	150 (29.88%)	314 (31.53%)	0.555	0.0165	317 (31.57%)	150 (29.88%)	167 (33.27%)	0.277	0.0339
Sepsis	154 (10.28%)	57 (11.35%)	97 (9.74%)	0.378	0.0162	113 (11.25%)	57 (11.35%)	56 (11.16%)	1	0.002
Copd	175 (11.68%)	51 (10.16%)	124 (12.45%)	0.223	0.0229	100 (9.96%)	51 (10.16%)	49 (9.76%)	0.916	0.004
Hypertension	491 (32.78%)	138 (27.49%)	353 (35.44%)	0.002	0.0795	279 (27.79%)	138 (27.49%)	141 (28.09%)	0.888	0.006
Acute_pancreatitis	35 (2.34%)	8 (1.59%)	27 (2.71%)	0.242	0.0112	16 (1.59%)	8 (1.59%)	8 (1.59%)	1	0
Acute renal failure	320 (21.36%)	91 (18.13%)	229 (22.99%)	0.036	0.0486	185 (18.43%)	91 (18.13%)	94 (18.73%)	0.871	0.006
Sofa	6.0 [4.0, 9.0]	7.0 [4.0, 10.0]	6.0 [4.0, 8.0]	< 0.001	0.2519	7.0 [4.0, 9.0]	7.0 [4.0, 10.0]	7.0 [5.0, 9.0]	0.842	0.0336
Sapsii	41.0 [33.0, 51.0]	41.0 [34.0, 51.0]	40.0 [32.0, 51.0]	0.045	0.1142	42.0 [34.0, 52.0]	41.0 [34.0, 51.0]	42.0 [34.0, 52.0]	0.982	0.0215
Oasis	36.0 [31.0, 42.0]	36.0 [31.0, 42.0]	36.0 [31.0, 42.0]	0.337	0.0473	36.0 [31.0, 42.0]	36.0 [31.0, 42.0]	36.0 [31.0, 42.0]	0.575	0.0306
Heart_rate_mean	87.38 [76.41, 99.36]	84.81 [75.57, 97.53]	88.91 [76.71, 100.08]	0.015	0.1034	86.66 [75.75, 98.56]	84.81 [75.57, 97.53]	88.74 [75.97, 98.88]	0.181	0.0468
mbp	75.31 [70.0, 82.65]	74.72 [69.53, 81.93]	75.60 [70.11, 83.09]	0.089	0.0679	74.59 [69.58, 81.90]	74.72 [69.53, 81.93]	74.56 [69.72, 81.88]	0.92	0.0331
Resp_rate	19.65 [17.27, 22.52]	18.83 [16.74, 21.82]	20.13 [17.61, 22.76]	< 0.001	0.2077	19.11 [16.98, 21.92]	18.83 [16.74, 21.82]	19.39 [17.20, 22.02]	0.106	0.0418
pao2fio2ratio	130.0 [82.89, 194.21]	136.67 [88.57, 206.5]	125.0 [80.0, 189.25]	0.01	0.146	136.67 [88.0, 208.08]	136.67 [88.57, 206.5]	136.67 [87.36, 209.64]	0.921	0.0057
Vaso	871 (58.14%)	313 (62.35%)	558 (56.02%)	0.022	0.0633	623 (62.05%)	313 (62.35%)	310 (61.75%)	0.897	0.006
Ventilation	661 (44.13%)	204 (40.64%)	457 (45.88%)	0.061	0.0525	416 (41.43%)	204 (40.64%)	212 (42.23%)	0.654	0.0159
rrt	171 (11.42%)	75 (14.94%)	96 (9.64%)	0.003	0.053	148 (14.74%)	75 (14.94%)	73 (14.54%)	0.929	0.004
crrt	119 (7.94%)	46 (9.16%)	73 (7.33%)	0.255	0.0183	98 (9.76%)	46 (9.16%)	52 (10.36%)	0.595	0.012

### Outcomes

Utilizing univariate Cox regression analysis and the Mann–Whitney *U* test to examine continuous and categorical variables, it was observed that patients receiving prophylactic heparin demonstrated a significantly lower in-hospital mortality rate compared to those who did not (13.55% vs 17.93%, HR = 0.71, 95% CI 0.54–0.93, *P* = 0.012). After propensity score matching (PSM), the association of heparin use with reduced in-hospital mortality remained significant (12.75% vs 17.93%, HR = 0.65, 95% CI 0.47–0.90, *P* = 0.010) (as depicted in Table 2). Regarding secondary outcomes, the analysis indicated that patients who received early heparin administration showed a significantly reduced mortality rate at 28 days, 60 days, and 90 days, in comparison to those not receiving heparin. Specifically, in the pre-matched cohort, early heparin use was associated with a significant reduction in the 28-day mortality rate (HR = 0.74; 95% CI 0.57–0.95; *P* = 0.019), a finding that was corroborated post-PSM (HR = 0.73; 95% CI 0.54–0.98; *P* = 0.037). Additionally, at 60 days, the heparin group also exhibited a significant reduction in mortality (HR = 0.72; 95% CI 0.58–0.90; *P* = 0.004), with PSM data supporting this finding (HR = 0.70; 95% CI 0.54–0.91; *P* = 0.009). For the 90-day mortality rate, the trend of reduction in the early heparin group was similarly significant (HR = 0.71; 95% CI 0.58–0.88; *P* = 0.002), and this result was again confirmed post-PSM (HR = 0.68; 95% CI 0.53–0.87; *P* = 0.002). However, the duration of ICU stay and total hospital stay were slightly longer in the heparin group compared to the non-heparin group, with ICU stay duration being (pre-matching: 5.13 days vs 4.29 days, *P* < 0.001; post-PSM: 5.0 days vs 4.29 days, *P* = 0.008) and total hospital stay (pre-matching: 13 days vs 12 days, *P* = 0.022; post-PSM: 13 days vs 12.0 days, *P* = 0.015) (as depicted in Table 2).

**Table 2:** Impact of prophylactic heparin on clinical outcomes in ARDS patients before and after propensity score matching

Outcome	Before PSM					After PSM				
	All patients (*n* = 1498)	Non-heparin (*n* = 502)	Early heparin users (*n* = 996)	Adjusted HR (95% CI)	*P*-value	All patients (*n* = 1004)	Non-heparin (*n* = 502)	Early heparin users (*n* = 502)	Adjusted HR (95% CI)	*P*-value
Length of ICU stay, mean (SD)	4.96 [3.13, 9.25]	4.29 [2.92, 8.24]	5.13 [3.33, 9.79]		< 0.001	4.79 [3.04, 8.93]	4.29 [2.92, 8.24]	5.0 [3.29, 9.16]		0.008
Length of hospital stay, mean (SD)	12.0 [7.0, 21.0]	12.0 [7.0, 20.0]	13.0 [8.0, 22.0]		0.022	12.0 [7.0, 21.0]	12.0 [7.0, 20.0]	13.0 [8.0, 22.0]		0.015
In-hospital mortality, *n* (%)	225 (15.02%)	90 (17.93%)	135 (13.55%)	0.71 (0.54–0.93)	0.012	154 (15.34%)	90 (17.93%)	64 (12.75%)	0.65 (0.47–0.90)	0.01
7-day mortality, *n* (%)	95 (6.34%)	41 (8.17%)	54 (5.42%)	0.66 (0.44–0.99)	0.044	70 (6.97%)	41 (8.17%)	29 (5.78%)	0.70 (0.43–1.12)	0.139
14-day mortality, *n* (%)	170 (11.35%)	63 (12.55%)	107 (10.74%)	0.83 (0.61–1.13)	0.238	119 (11.85%)	63 (12.55%)	56 (11.16%)	0.85 (0.60–1.23)	0.395
28-day mortality, *n* (%)	256 (17.09%)	100 (19.92%)	156 (15.66%)	0.74 (0.57–0.95)	0.019	179 (17.83%)	100 (19.92%)	79 (15.74%)	0.73 (0.54–0.98)	0.037
60-day mortality, *n* (%)	329 (21.96%)	129 (25.70%)	200 (20.08%)	0.72 (0.58–0.90)	0.004	229 (22.81%)	129 (25.70%)	100 (19.92%)	0.70 (0.54–0.91)	0.009
90-day mortality, *n* (%)	370 (24.70%)	146 (29.08%)	224 (22.49%)	0.71 (0.58–0.88)	0.002	256 (25.50%)	146 (29.08%)	110 (21.91%)	0.68 (0.53–0.87)	0.002

**Fig. 3:**
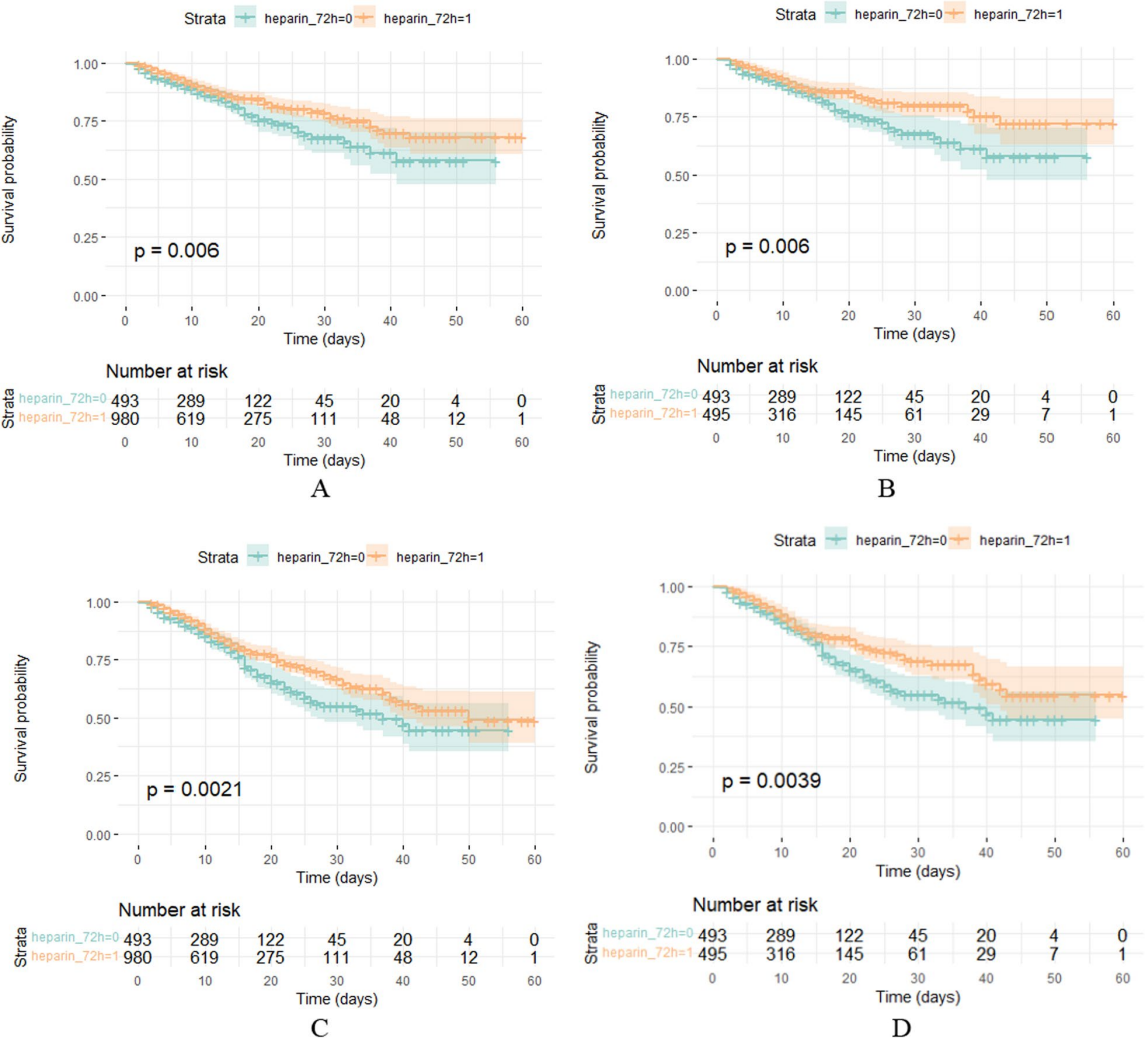
Survival analysis of heparin and non-heparin groups. Kaplan–Meier survival curves for the in-hospital (**A**, **B**), 60-day (**C**, **D**) mortality among all patients are shown. Kaplan–Meier survival curves for pre-matched cohort (**A**, **C**) and matched cohort (**B**, **D**)

Kaplan–Meier survival curves and log-rank tests further confirmed the benefits of early heparin use (as shown in Fig. 3). An expanded multivariable Cox regression model was employed to assess the impact of early heparin use on patient outcomes. The effect of significantly reduced mortality risk associated with early heparin administration persisted even after adjusting for multiple covariates (see Table 3 for details). Specifically, it was found that patients receiving 5–6 doses of heparin within the first 72 h exhibited a significantly lower risk of mortality within 60 days (HR = 0.541, 95% CI 0.349–0.838, *P* = 0.006), as described in Table 4.

**Table 3:** Correlation of early heparin use with in-hospital mortality in an ARDS cohort

Variables	Before PSM			After PSM		
	HR	(95% CI)	*P*-value	HR	(95% CI)	*P*-value
Model 1	0.714	0.545–0.936	0.015	0.643	0.466–0.889	0.007
Model 2	0.741	0.563–0.975	0.032	0.638	0.462–0.882	0.007
Model 3	0.711	0.539–0.938	0.016	0.646	0.467–0.894	0.008
Model 4	0.701	0.531–0.925	0.012	0.636	0.459–0.881	0.006
Model 5	0.706	0.536–0.930	0.013	0.630	0.455–0.873	0.005

Adjusted covariates: Model 1 = gender + age at admission + ethnicity + comorbidities (cancer, diabetes, sepsis, chronic obstructive pulmonary disease, hypertension, acute pancreatitis, acute renal failure) Model 2 = Model 1 + clinical scoring systems(Sequential Organ Failure Assessment score measured within 24 h, Simplified Acute Physiology Score II, Oxford Acute Severity of Illness Score) Model 3 = Model 2 + vital signs upon admission (mean heart rate, mean blood pressure, mean respiratory rate, minimum PaO2/FiO2 ratio at diagnosis) Model 4 = Model 3 + treatment interventions (use of vasopressors, mechanical ventilation, RRT, CRRT) Model 5 (Lasso Regression Selected Variables) = gender + age at admission + cancer + diabetes + sepsis + acute pancreatitis + acute renal failure + Sequential Organ Failure Assessment score measured within 24 h + Simplified Acute Physiology Score II + Oxford Acute Severity of Illness Score + mean heart rate + mean respiratory rate + mechanical ventilation + CRRT + mean blood pressure

**Table 4:** Correlation of early heparin use with patient 60-day mortality in an ARDS cohort

Variables	Before PSM			After PSM		
	HR	(95% CI)	*P*-value	HR	(95% CI)	*P*-value
Model 1	0.735	0.587–0.920	0.007	0.695	0.534–0.904	0.007
Model 2	0.753	0.600–0.946	0.015	0.691	0.531–0.900	0.006
Model 3	0.699	0.555–0.881	0.002	0.692	0.531–0.902	0.006
Model 4	0.699	0.554–0.881	0.002	0.700	0.537–0.912	0.008
Model 5	0.736	0.586–0.924	0.008	0.693	0.533–0.903	0.006
Dose 1–2	0.913	0.653–1.277	0.595	0.685	0.415–1.131	0.139
Dose 3–4	1.125	0.851–1.488	0.408	1.330	0.947–1.868	0.100
Dose 5–6	0.719	0.532–0.971	0.032	0.541	0.349–0.838	0.006
Dose 7–8	0.863	0.612–1.216	0.399	0.644	0.371–1.118	0.118
Dose 9–10	0.586	0.287–1.198	0.143	0.833	0.307–2.260	0.719

Adjusted covariates: Model 1 = gender + age at admission + ethnicity + comorbidities (cancer, diabetes, sepsis, chronic obstructive pulmonary disease, hypertension, acute pancreatitis, acute renal failure) Model 2 = Model 1 + clinical scoring systems(Sequential Organ Failure Assessment score measured within 24 h, Simplified Acute Physiology Score II, Oxford Acute Severity of Illness Score) Model 3 = Model 2 + vital signs upon admission (mean heart rate, mean blood pressure, mean respiratory rate, minimum PaO2/FiO2 ratio at diagnosis) Model 4 = Model 3 + treatment interventions (use of vasopressors, mechanical ventilation, RRT, CRRT) Model 5 (Lasso Regression Selected Variables) = gender + age at admission + cancer + diabetes + sepsis + chronic obstructive pulmonary disease + acute pancreatitis + acute renal failure + Sequential Organ Failure Assessment score measured within 24 h + Simplified Acute Physiology Score II + Oxford Acute Severity of Illness Score + Mean heart rate + mean respiratory rate + use of vasopressors + mechanical ventilation

Furthermore, for patients with ARDS, early heparin use significantly decreased the risk of in-hospital, 60-day, and 90-day mortality. This conclusion is supported not only in traditional multivariate analysis, but also validated through dual robust analysis and Inverse Probability Weighting (IPW) methods, demonstrating strong consistency in results (see Table 5).

**Table 5:** Comparative analysis of mortality in ARDS patients across five statistical models

	HR	CI (2.5%)	CI (97.5%)	*P* value
In-hospital mortality				
Doubly robust with unbalanced covariates	0.669	0.487	0.919	0.013
Doubly robust with all covariates	0.705	0.515	0.965	0.029
PS (IPW)	0.660	0.491	0.888	0.006
PS matching	0.650	0.470	0.900	0.010
Multivariate	0.706	0.536	0.930	0.013
60-day mortality				
Doubly robust with unbalanced covariates	0.664	0.509	0.865	0.002
Doubly robust with all covariates	0.707	0.544	0.918	0.009
PS (IPW)	0.695	0.544	0.888	0.004
PS matching	0.700	0.540	0.910	0.009
Multivariate	0.736	0.586	0.924	0.008
90-day mortality				
Doubly robust with unbalanced covariates	0.652	0.508	0.836	0.001
Doubly robust with all covariates	0.685	0.535	0.877	0.003
PS (IPW)	0.684	0.544	0.861	0.001
PS matching	0.680	0.530	0.870	0.002
Multivariate	0.711	0.574	0.882	0.002

### Subgroup analysis

**Fig. 4:**
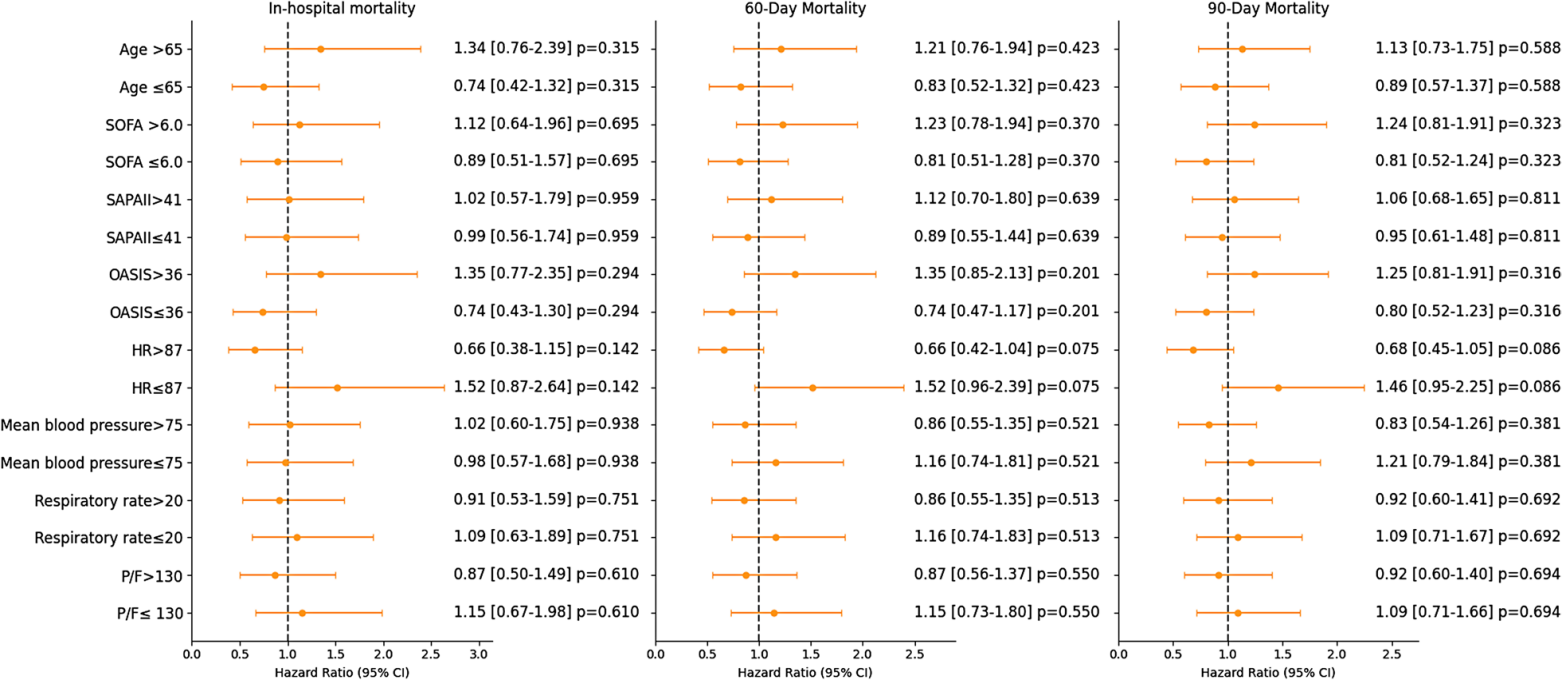
Subgroup analysis of the effect of early heparin used on mortality rates in ARDS patients

Subgroup analysis showed no significant difference in the effect of early heparin treatment on mortality rates across different subgroups (as depicted in Fig. 4).

### Sensitivity analysis

Sensitivity analysis began with variable selection via Lasso regression, followed by the application of these variables in multivariate Cox proportional hazards modeling to evaluate risk factors for in-hospital mortality (as shown in Table 6). *E*-value analysis indicated that an observed association between heparin and in-hospital mortality would require an HR greater than 2.18 to be explained by unmeasured confounding factors (as shown in Table 7). This suggests that, even after accounting for known risk factors such as tumors, the significant impact of other unknown or unmeasured factors on mortality is relatively small. Combined with Additional file 1: Tables S1, Additional file 2: Table S2, it can be seen that other unknown or unmeasured factors also have a relatively small effect on 60-day, 90-day mortality.

**Table 6:** Selection of risk variables for in-hospital mortality in patients with ARDS using Lasso regression and Cox proportional hazards analysis

Variables	HR	95%CI	*P* value
Gender	0.767	0.582–1.012	0.061
Ventilation	1.166	0.886–1.533	0.273
crrt	1.222	0.785–1.902	0.376
Cancer	1.357	0.961–1.916	0.083
Diabetes	0.817	0.600–1.111	0.198
Sepsis	1.101	0.748–1.619	0.626
Acute_pancreatitis	0.510	0.206–1.262	0.145
ARF	1.118	0.821–1.523	0.478
Admission_age	1.039	1.027–1.050	0.000
Sofa_24hours	1.068	1.008–1.132	0.027
Sapsii	0.988	0.971–1.005	0.163
Oasis	1.012	0.988–1.038	0.328
Heart_rate_mean	1.002	0.993–1.011	0.707
mbp_mean	1.004	0.990–1.019	0.562
Resp_rate_mean	1.059	1.024–1.095	0.001
Heparin_72h	0.706	0.536–0.930	0.013

**Table 7:** *E*-value for mortality association in heparin-treated ARDS patients

Outcomes	*E*-value	Upper limit of 95% CI
In-hospital mortality	2.18	1.36
60-day mortality	2.06	1.38
90-day mortality	2.16	1.52

### Further analysis

To evaluate the impact of different forms of heparin therapy administered within the first 72 h on in-hospital mortality rates, we compared the effects of subcutaneous administration of prophylactic doses of heparin with those of intravenous bolus or infusion of therapeutic doses of heparin. To further analyze the differences between these two treatment modalities, we incorporated the relevant data into a multifactorial Cox regression model for evaluation. The analysis revealed that a significant reduction in in-hospital mortality rates was observed only in the context of subcutaneous administration of prophylactic doses of heparin (see Table 8 for details).

**Table 8:** Comparison of in-hospital mortality impact between subcutaneous prophylactic heparin and intravenous therapeutic heparin in the initial 72 hours

Variables	HR	95%CI	*P* value
In-hospital mortality			
heparin_sc72h_only	0.568	0.372–0.867	0.009
heparin_push72h_only	0.000	0–0	1.000
heparin_iv72h_only	0.542	0.233–1.26	0.155
no_heparin_72h	0.707	0.456–1.098	0.123
60-day mortality			
heparin_sc72h_only	0.614	0.428–0.883	0.008
heparin_push72h_only	0.000	0–0	1.000
heparin_iv72h_only	0.565	0.284–1.123	0.103
no_heparin_72h	0.746	0.512–1.087	0.127
90-day mortality			
heparin_sc72h_only	0.640	0.448–0.913	0.014
heparin_push72h_only	0.261	0.036–1.911	0.186
heparin_iv72h_only	0.587	0.303–1.138	0.115
no_heparin_72h	0.787	0.545–1.138	0.203

## Discussion

The main finding of this study is that early use of prophylactic doses of heparin is associated with reduced in-hospital and long-term mortality in patients with ARDS, but may slightly prolong the duration of hospital stays. ARDS, a clinical emergency with high mortality rates, is currently managed with supportive treatments aimed at preventing the exacerbation of lung injury and improving outcomes, such as mechanical ventilation, prone positioning, neuromuscular blockade, and extracorporeal life support [23]. Yet, new therapies targeting the pathophysiology of ARDS development are urgently needed. Heparin, as a potential treatment option, not only garners attention for its anticoagulant effects but also for its anti-inflammatory properties. In the complex pathophysiology of ARDS, which involves increased inflammation and procoagulant factors and the destruction of the alveolar–capillary barrier, heparin's multifunctionality could offer a novel therapeutic perspective [24].

Early intervention with heparin is crucial for ARDS treatment strategies, possibly due to its impact on the inflammatory response and the progression of secondary injury, as well as its role in reducing the risk of microvascular thrombosis, thereby helping to maintain the stability of the pulmonary microcirculation and improve clinical outcomes for patients [25]. Our findings echo those of existing studies. For instance, a meta-analysis demonstrated that heparin significantly reduced the 28-day mortality rate in patients with severe sepsis [26]. Furthermore, an open-label, adaptive, multi-platform, controlled trial found that a therapeutic dose anticoagulation strategy with heparin not only improved the probability of survival upon discharge but also reduced the need for cardiovascular or respiratory organ support [27]. Nevertheless, some studies have indicated that the use of therapeutic doses of heparin did not reduce mortality in patients with Acute Lung Injury (ALI), suggesting that the benefits of heparin may vary across different patient groups and conditions [28].

A retrospective, propensity score-matched, multicenter cohort study that found heparin use was associated with a significant decrease in death within 28 days, especially as the disease severity increased, lends additional support to our findings. The use of heparin was also associated with successful weaning from mechanical ventilation and a reduction in the use of vasopressor and muscle strength support medications, findings that were especially pronounced in the common ARDS complication of septic shock [29]. Moreover, there is an association between heparin treatment and improved prognosis in hospitalized patients with COVID-19, suggesting its potential for broad application in clinical treatment [30]. In a pivotal observational cohort study, researchers evaluated the clinical outcomes of COVID-19 patients who initiated early prophylactic anticoagulation treatment versus those who did not receive such treatment. The results indicated that, in the group of patients who received prophylactic anticoagulation therapy, the risk of death within 30 days was reduced by 27% compared to patients who did not receive this treatment [31]. Furthermore, for critically ill COVID-19 patients, early therapeutic anticoagulation treatment did not significantly improve in-hospital survival rates compared to patients who did not receive such treatment [32].

Current literature has investigated the role of circulating extracellular histones in exacerbating pulmonary endothelial dysfunction and acute lung injury (ALI), and has demonstrated the protective effect of heparin in inhibiting histone-induced inflammation [33]. Additionally, the method of nebulized administration highlights the potential benefits of heparin for the treatment of ARDS, especially in the context of COVID-19-related ARDS, as observed in animal models [34] and human studies [14, 35–37]. These findings may reflect the dual action of heparin's anti-inflammatory and anticoagulant functions and its particular effectiveness in treating pulmonary injury caused by viral diseases.

In delving into the potential mechanisms of heparin, our study has revealed its association with reduced mortality rates in ARDS patients, which may be partly attributable to its pathophysiological roles. Heparin possesses a spectrum of anti-inflammatory, mucolytic, and antimicrobial pharmacological properties that can mitigate various aspects of the inflammatory response, including endothelial adhesion, leukocyte migration and activation, as well as the neutralization of released tissue-damaging mediators [38]. Heparin notably ameliorates LPS-induced pulmonary cell injury by inhibiting the expression of pro-inflammatory cytokines and the NF-κB pathway within macrophages [39]. Serving as an effective inflammatory modulator, heparin suppresses the expression and function of adhesion molecules, demonstrating its anti-inflammatory activity in vivo [40, 41]. Additionally, heparin directly modulates pro-inflammatory mediators, such as inhibiting p38 MAPK and NF-κB activation, and alleviates endothelial cell dysfunction through the nitric oxide system, significantly preventing endothelium-mediated immune responses [42, 43]. The cumulative effects of these actions may contribute to reducing the inflammatory burden in ARDS patients, improving pulmonary function, and ultimately decreasing mortality rates.

In conclusion, heparin's multiple actions not only alter the molecular pathways of inflammation and coagulation in ARDS but also demonstrate potential in improving clinical outcomes for patients. These findings provide us with a biological rationale for the use of heparin in the treatment of ARDS and may explain the clinical benefits observed in our cohort study.

In the current literature, studies exploring the impact of subcutaneously administered prophylactic heparin on acute respiratory distress syndrome (ARDS) are relatively scarce. The majority of related research has focused on the effects of therapeutically dosed heparin, administered via nebulization or intravenous injection, on ARDS patients, particularly in those studies concerning COVID-19 patients [25, 44]. In contrast, our study delves into the potential benefits of early subcutaneous administration of prophylactic heparin in non-COVID-19 ARDS patients. Presently, there is no expert consensus on the use of heparin in ARDS treatment. However, our research provides crucial preliminary evidence suggesting that the early application of prophylactic doses of heparin may be associated with a reduction in early mortality rates in ARDS patients. Through multifactorial Cox regression analysis, our study discovered that administering 5–6 subcutaneous injections of heparin sodium within the first 72 h can significantly lower the 60-day mortality rate in ARDS patients. This finding resonates with the latest guidelines released in 2021 by the International Society on Thrombosis and Haemostasis (ISTH) for COVID-19 patients, which recommend the preference of prophylactic doses (UFH 5000 U SQ BID or TID) over moderate doses of low molecular weight heparin or standard heparin, to reduce the risk of adverse events (including death and thrombosis formation) in critically ill COVID-19 patients [45]. Our study offers a new perspective in the treatment of ARDS and may have significant implications for clinical practice.

Nevertheless, as a single-center retrospective study, our research has certain limitations, including the representativeness of the sample, challenges with data integrity and accuracy, and the difficulty in controlling for selection bias and confounding factors. Although statistical methods such as propensity score matching and IPW were used to mitigate these issues, residual confounding factors not included in the analysis may still exist.

Future research should validate our findings through multicenter, prospective studies and pinpoint the precise role of heparin therapy in the management of ARDS. Furthermore, larger-scale randomized controlled trials are warranted to refine the dosing, mode of administration, and effects of heparin across different ARDS patient subtypes to optimize its clinical application strategy.

## Conclusion

This research highlights that early prophylactic administration of heparin may substantially lower mortality in ARDS patients. These findings underscore the potential of heparin as a key component in the management of ARDS, offering a new perspective and novel strategies for clinical treatment.

### Supplementary Information

**Additional file 1: Table S1. **Selection of Risk Variables for 60-Day Mortality in ARDS Patients Using Lasso Regression Followed by Cox Proportional Hazards Analysis.

**Additional file 2: Table S2. **Selection of Risk Variables for 90-Day Mortality in ARDS Patients Using Lasso Regression Followed by Cox Proportional Hazards Analysis.

## Data Availability

The data that support the fundings of this study are available from the MIMICIV database, but restrictions apply to the availability of these data, which were used under license for the current research and so are not publicly available. Data are, however, available from the authors upon reasonable request and with permission of the holder of the database.

## Abbreviations

ARDS: Acute respiratory distress syndrome
PSM: Propensity score matching
SOFA: Sequential Organ Failure Assessment
SAPS II: Simplified Acute Physiology Score II
OASIS: Oxford Acute Severity of Illness Score
COPD: Chronic obstructive pulmonary disease
RRT: Renal replacement therapy
CRRT: Continuous renal replacement therapy
IPW: Inverse probability weighting
GBM: Gradient boosting models
ALI: Acute lung injury
BIDMC: Beth Israel Deaconess Medical Center
MIMIC-IV: Medical Information Mart for Intensive Care IV
PEEP: Positive end-expiratory pressure
