# Supplementary material for: Exploring the therapeutic role of early heparin administration in ARDS management: a MIMIC-IV database analysis
Source: J Intensive Care. 2024 Feb 26;12:9. doi: 10.1186/s40560-024-00723-5 (PMC10895755; doi:10.1186/s40560-024-00723-5)
Supplement: Supplementary file 1 — Additional file 1: Table S1. Selection of Risk Variables for 60-Day Mortality in ARDS Patients Using Lasso Regression Followed by Cox Proportional Hazards Analysis. [file 40560_2024_723_MOESM1_ESM.docx]

Table S1: Selection of Risk Variables for 60-Day Mortality in ARDS Patients Using Lasso Regression Followed by Cox Proportional Hazards Analysis.

| Variables | HR | 95％CI | P value |
| --- | --- | --- | --- |
| gender | 0.773 | 0.616 - 0.971 | 0.027 |
| vaso | 0.854 | 0.658 - 1.109 | 0.237 |
| ventilation | 1.002 | 0.799 - 1.256 | 0.986 |
| cancer | 1.547 | 1.175 - 2.038 | 0.002 |
| diabetes | 0.960 | 0.752 - 1.226 | 0.744 |
| sepsis | 1.178 | 0.865 - 1.604 | 0.298 |
| copd | 1.249 | 0.910 - 1.715 | 0.169 |
| acute_pancreatitis | 0.316 | 0.116 - 0.855 | 0.023 |
| ARF | 1.015 | 0.784 - 1.314 | 0.908 |
| admission_age | 1.036 | 1.026 - 1.046 | 0.000 |
| sofa_24hours | 1.056 | 1.006 - 1.108 | 0.029 |
| sapsii | 0.994 | 0.980 - 1.008 | 0.387 |
| oasis | 1.010 | 0.989 - 1.030 | 0.359 |
| heart_rate_mean | 1.001 | 0.993 - 1.008 | 0.879 |
| resp_rate_mean | 1.044 | 1.015 - 1.074 | 0.003 |
| heparin_72h | 0.736 | 0.586 - 0.924 | 0.008 |
