# Supplementary material for: Exploring the therapeutic role of early heparin administration in ARDS management: a MIMIC-IV database analysis
Source: J Intensive Care. 2024 Feb 26;12:9. doi: 10.1186/s40560-024-00723-5 (PMC10895755; doi:10.1186/s40560-024-00723-5)
Supplement: Supplementary file 2 — Additional file 2: Table S2. Selection of Risk Variables for 90-Day Mortality in ARDS Patients Using Lasso Regression Followed by Cox Proportional Hazards Analysis. [file 40560_2024_723_MOESM2_ESM.docx]

Table S2: Selection of Risk Variables for 90-Day Mortality in ARDS Patients Using Lasso Regression Followed by Cox Proportional Hazards Analysis.

| Variables | HR | 95％CI | P value |
| --- | --- | --- | --- |
| gender | 0.779 | 0.629 - 0.966 | 0.023 |
| vaso | 0.848 | 0.663 - 1.085 | 0.190 |
| ventilation | 1.005 | 0.812 - 1.245 | 0.961 |
| rrt | 0.701 | 0.493 - 0.997 | 0.048 |
| cancer | 1.471 | 1.132 - 1.911 | 0.004 |
| diabetes | 0.959 | 0.760 - 1.211 | 0.725 |
| sepsis | 1.208 | 0.906 - 1.612 | 0.198 |
| acute_pancreatitis | 0.467 | 0.218 - 0.999 | 0.050 |
| ARF | 0.985 | 0.771 - 1.259 | 0.904 |
| admission_age | 1.033 | 1.024 - 1.042 | 0.000 |
| sofa_24hours | 1.055 | 1.007 - 1.105 | 0.023 |
| sapsii | 0.994 | 0.981 - 1.008 | 0.401 |
| oasis | 1.013 | 0.993 - 1.032 | 0.201 |
| heart_rate_mean | 0.999 | 0.992 - 1.006 | 0.835 |
| resp_rate_mean | 1.039 | 1.012 - 1.068 | 0.005 |
| heparin_72h | 0.711 | 0.574 - 0.882 | 0.002 |
